# India’s disability estimates: Limitations and way forward

**DOI:** 10.1371/journal.pone.0222159

**Published:** 2019-09-06

**Authors:** Rakhi Dandona, Anamika Pandey, Sibin George, G. Anil Kumar, Lalit Dandona

**Affiliations:** 1 Public Health Foundation of India, Gurugram, National Capital Region, India; 2 Institute for Health Metrics and Evaluation, University of Washington, Seattle, Washington, United states of America; Università degli Studi di Perugia, ITALY

## Abstract

**Background:**

With India preparing for the next decennial Census in 2021, we compared the disability estimates and data collection methodology between the Census 2011 and the most recent population-level survey for India and its states, to highlight the issues to be addressed to improve robustness of the disability estimates in the upcoming Census.

**Methods:**

Data from the Census 2011 and from two complementary nationally representative household surveys that covered all Indian states with the same methodology and survey instruments–the District-Level Household Survey-4 (DLHS-4, 2012–2013) and the Annual Health Surveys (AHS three rounds, 2010–11, 2011–12 and 2012–13) were used. Data from DLHS-4 and AHS 2012–13 round were pooled to generate estimates for the year 2012–13. Data collection methodology between the sources was compared, including the review of definitions of each type of disability. The overall, mental, visual, hearing, speech, and movement disability rate (DR) per 100,000 population were compared between the sources for India and for each state, and the percent difference in the respective rates was calculated. We explored the reliability of these estimates comparing yearly data from the AHS for three successive rounds.

**Results:**

Survey data were collected through proxy reporting, however, it is not entirely clear whether the data were proxy- or self-reported or a mix of both in the Census. The overall DR was 25.1% higher in the Census (2,242; 95% CI 2,241–2,243) than the survey (1,791; 95% CI 1,786–1,797) per 100,000 population, with the state-level difference ranging from -64% in Tamil Nadu to 107% in Sikkim state. Despite both sources using nearly similar definitions for overall disability and disability by type, the difference in DR was 125.5%, 54.2%, -25.7%, -19.7%, and 21.9% for hearing, speech, mental, movement, and visual DR, respectively. At the state-level, the difference in disability-specific estimates ranged from -84% to 450%. The extent of variations in the disability-specific estimates in AHS successive rounds ranged from -25% to 929% at the state-level.

**Conclusions:**

There is momentum globally towards building disability measurement that is consistent with the data required for monitoring of the Sustainable Development Goals to ensure robust estimation of disability. The current estimates from the Census and surveys seem much lower than would be expected at the population level. We make recommendations that India needs to take serious note of in order to improve the validity and reliability of India’s disability estimates.

## Introduction

The global commitment for the 2030 Agenda for Sustainable Development recognizes the promotion of the rights, perspectives and well-being of persons with disabilities in line with the Convention of the Rights of Persons with Disabilities (CRPD) towards a more sustainable and inclusive world [1–3]. According to the World Report on Disability released in 2011, nearly 15% of the world’s population lived with some form of disability, of whom 2–4% experienced significant difficulties in functioning [4]. It also highlighted that the prevalence of disability was on the rise due to ageing populations and the higher risk of disability in older people as well as the global increase in chronic health conditions [4]. The World Report was based on best available prevalence data from the World Health Surveys, country reported Census and survey data, and data from the Global Burden of Disease (GBD) Study [4]. The GBD Study provides estimates for disability on an on-going basis from more than 350 diseases/conditions in 195 countries from 1990 onward using a variety of data sources including disease/condition specific clinical and self-reported surveys [5]. One of the key recommendations of the World Report was for the nations to improve disability data collection, as major shortcomings of the national disability prevalence data were highlighted, in particular underestimation and lack of comparability [4, 6]. The recent United Nations Flagship Report on Disability and Development 2018 also highlights the need to significantly increase the availability of high-quality, timely and disaggregated data by disability as one of the major requirements to monitor progress made for persons with disabilities [1]. This need for availability of relevant data is in line with the CRPD which calls on States Parties to collect appropriate information, including statistical and research data, to enable them to formulate and implement policies related to the Convention [2].

Disability is complex and multi-faceted, with some of its roots in society and culture [7]. Therefore, counting disability is a challenge, and various issues in disability measurement between and within countries due to varying definitions, data collection systems, age range and populations included are known [8–12]. To address these challenges, globally there is a momentum towards building disability measurement that is consistent with the Sustainable Development Goals which outline the major goals of policy formulation and program planning with the aim to promote participation of persons with disabilities in all aspects of life [13]. Advances such as the United Nation’s Washington Group Statistics short set (WGSS) are being made to ensure the comparability of disability estimates in the Censuses and household surveys globally, as the WGSS is consistent with interactional understanding of disability, i.e. disability resulting from the interaction of an individual with a health condition or impairment with the environment, for instance as in the International Classification of Functioning, Disability and Health [6, 14–16].

As is in most developing countries, India uses the disability estimates from the Census to inform policy and programs for persons with disabilities [17–21]. In the most recent Census of 2011, disability information became more detailed and important, and included information on a wide range of disabilities [21, 22]. According to this latest Census 2011, 2.2% of the Indian population had disability [20, 21]. Disability data in India are also available from large-scale population surveys such as the National Sample Survey, District-Level Household Survey-4, the Annual Health Survey, and the World Health Survey [23–28]. A previous comparison noted wide variations in the disability estimates from the Census 2001 and the National Sample Survey of persons with disabilities conducted in 2002 [27, 29], and the need for more qualitative studies on disability has been argued. As India prepares for the next decennial Census in the context of its commitment to SDGs and CRPD, we aim to provide a review of the disability estimates for India and its states from the Census 2011 and the most recent population-level survey estimates for disability in the current decade. In the background of the above stated need for high-quality data on disability, it is important to understand the variations across the data sources in India in order to provide a consistent and reliable information to formulate and implement policies for people with disability and to monitor trends in the prevalence of disabilities. Therefore, this paper aims to highlight the extent of variation in the disability estimates, and the methodological and definition related issues between the data sources that provide disability estimates for India for all age groups, and to recommend ways in which these variations could be minimized to reliably monitor the trends in disability over time.

## Methods

The data sources used for this analysis are shown in Table 1. The latest decennial population Census of 2011 [21] aimed to provide a complete coverage of all persons with disabilities for all ages in all states of India by including all types of disabilities listed under the Persons with Disability Act, 1995 and the National Trust Act, 1999 [30, 31]. The other data sources in the current decade that provided data in public domain on disability for all age groups were two complementary nationally representative household surveys–the District-Level Household Survey-4 (DLHS-4, 2012–2013) and the Annual Health Survey (AHS, 2010–11, 2011–12 and 2012–13) [23–26]. DLHS was conducted by the International Institute for Population Sciences with an oversight by the Ministry of Health and Family Welfare, Government of India whereas the AHS was implemented by the Office of Registrar General of India. Both these surveys were done in coordination with the same methodology and survey instruments. DLHS-4 was conducted in all Indian states except the nine states that were covered by the AHS, which included the states of Bihar, Chhattisgarh, Jharkhand, Madhya Pradesh, Odisha, Rajasthan, Uttar Pradesh, Uttarakhand, and Assam [32]. Additionally, the AHS was also a panel survey in which the households surveyed in the baseline round (2010–11) were followed in the 1^st^ (AHS 2011–12) and 2^nd^ (AHS 2012–13) updation rounds [24–26].

**Table 1:** Data sources used in the assessment of disability prevalence in India.

Type of data source	Data source	Data collection period	Year for which data were collected	Indian states covered in the data source
Census	Census 2011	April 2010-September 2010 (1st phase), February 2011-March 2011 (2nd phase)	2010	All states
Survey	Annual Health Survey (Baseline)	July 2010-March 2011	2010	Assam, Bihar, Chhattisgarh, Jharkhand, Madhya Pradesh, Odisha, Rajasthan, Uttar Pradesh, Uttarakhand
	Annual Health Survey (First updation)	October 2011-April 2012	2011	
	Annual Health Survey (Second updation)	November 2012- May 2013	2012	
	District Level Household Survey-4	December 2012-March 2014	2012–2013	All states other than those covered in the Annual Health Survey (Andhra Pradesh, Arunachal Pradesh, Goa, Haryana, Himachal Pradesh, Karnataka, Kerala, Maharashtra, Manipur, Meghalaya, Mizoram, Nagaland, Punjab, Sikkim, Tamil Nadu, Telangana, Tripura, West Bengal)

We assessed the data collection methodologies across the data sources to document the universe of population covered, who provided the data on disability at the household level (head of the household or the individual member with disability), and we reviewed the key questions that were asked to document disability. A detailed review of the definitions of disability–overall and by each type–was done for the Census 2011 and household surveys. We reviewed the Census and the survey manuals to understand the operationalisation of the disability related questions [33, 34]. The types of disability assessed included mental, visual, hearing, speech, movement, multiple, and others.

We calculated the disability rate (DR) as the number of people with disability per 100,000 population from each data source. The overall DR and that by each type of disability was compared between the data sources to assess the validity of these estimates. We pooled the data from DLHS-4 and AHS round 2 (same survey time period as DLHS-4) to generate the DR for India for 2012–13 from household survey. We report the DR estimates for India and for each state from all the data sources, including the percent difference in the respective rates between the data sources. For India DR, we report the rate without the states/Union Territories of Delhi, Gujarat, Jammu and Kashmir, Dadra and Nagar Haveli, Daman and Dui, and Lakshadweep as data for these were not available from the household surveys. As the state of Telangana was created in 2014 out of Andhra Pradesh, data for the states of Andhra Pradesh and Telangana were separated by their districts in Census 2011 to produce separate DR for these two states for its comparison with household surveys. Furthermore, as three rounds of AHS data were available over a period of three years, we compared the overall DR and that by type within the subsequent rounds of the AHS in the nine states to explore the reliability of these estimates.

All analyses were done in STATA V.13.0 (StataCorp, College Station, Texas, USA) and Microsoft Excel 2016. As this analysis was based on secondary data available in public domain without personal identifiers, no ethics approval was needed.

## Results

### Data collection methods

#### Population universe

The Census collected data from individuals residing in “normal”, “institutional” and “houseless” households, whereas the household surveys collected data from individuals residing in “normal” households. Both the data sources captured information for all ages.

#### Type of respondent

Table 2 documents the respondent for the disability information across the data sources. In Census 2011, the questions on disability were asked from one member of the household during the population enumeration phase. The Census manual states that every possible effort is to be made to seek information on disability from the person with disability herself/himself, if she/he was present at the time of Census and was able to provide information. However, it is not possible to know what proportion of the data were proxy and self-reported. In the household surveys, the information on disability was collected from the head of the household or an adult respondent. Therefore, disability data was collected through proxy reporting in the surveys, however, it is not entirely clear whether the data were proxy-reported or self-reported or a mix of both in the Census 2011.

**Table 2:** Type of respondent, definition of disability and questions to document disability in the data sources used to assess prevalence of disability in India.

Data source	Type of respondent	Age group	Definition of disability	Questions to document disability
Census 2011	Member of the household /disabled person with disability	All ages	A physical or mental impairment that significantly restricts one or more major life activities.	(a) Is this person mentally/physically disabled?
				(b) If yes in (a), give code from the list below.
				In seeing-1
				In hearing-2
				In speech-3
				In movement-4
				Mental retardation-5
				Mental illness-6
				Any other-7
				Multiple disability-8
				(c) If multiple disability (Code ‘8’) in (b), give maximum three codes from the list above.
				This question was asked in respect of all members of the household.
Annual Health Survey (all rounds)	Head of the household/adult member of the household	All ages	A physical or mental condition that limits a person’s movements, senses, or activities.	Whether the household member has any form of disability as on the date of survey?
				Mental-1
				Visual-2
				Hearing-3
				Speech-4
				Movement-5
				Multiple-6
				Other*-7
				No disability-0
District Level Household Survey-4	Head of the household/adult member of the household	All ages	A physical or mental condition that limits a person’s movements, senses, or activities.	Whether the household member has any form of disability as on the date of survey?
				Mental-1
				Visual-2
				Hearing-3
				Speech-4
				Movement-5
				Multiple-6
				Other-7
				No disability-0
				0.
				0.

*This category was not available in Annual Health Survey Baseline (2010–11).

#### Questions to document disability

The Census 2011 had a screening question before asking for the type of disability whereas the surveys had only a single question to document disability (Table 2). Both Census and survey manuals provided instructions to the interviewers to explain to the respondent the actual purpose of the question and emphasize that the information on the number and type of disability would help the government in planning for the welfare of the people with disability. The Census manual in addition mentions that this information will help in taking adequate measures to provide equal opportunities in education and employment for people with disabilities, and will help in making public transportation, health services accessible to them. Even though the AHS question is phrased as “any form of disability” without specifically using the terms “physical or mental disability”, the operationalization of the question appears to be similar to the Census question as the survey manual instructs the interviewer to find out from the respondent if any member is suffering from any physical or mental disability.

### Overall disability

**Fig 1:**
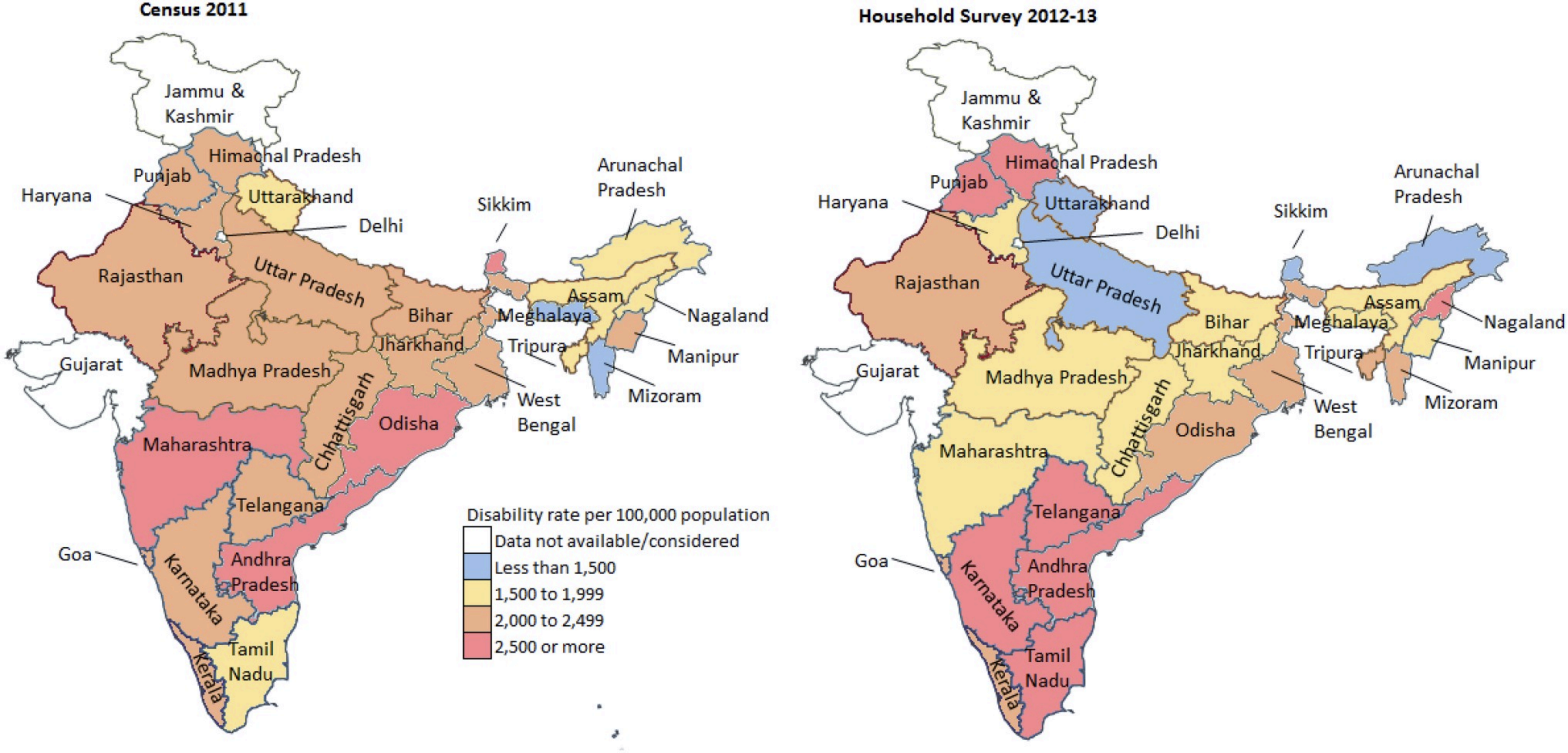
Disability rate in the Census 2011* and household survey 2012–2013^†‡^ for the Indian states. *Andhra Pradesh and Telangana were separated by the districts in the Census 2011. ^†^Includes pooled data from the District Level Household Survey-4 (2012–13) and Annual Health Survey 2^nd^ updation round (2012–13). ^‡^Data not available in the household survey for Delhi, Gujarat, Jammu and Kashmir, Dadra and Nagar Haveli, Daman and Dui, and Lakshadweep.

Both the data sources used nearly similar definition for overall disability in which disability is defined as a certain physical or mental impairment that resulted in restricted movement or senses or activity (S1 Table). The DR in Census 2011 (2,241.9; 95% CI 2,241.1–2,242.8) per 1,000 population was 25.1% higher than that in the surveys in 2012–13 (1,791.4; 95% CI 1,785.9–1,796.9) as shown in Table 3. Substantial variations at the state level were seen in DR between the sources ranging from -64% in Tamil Nadu to 107% in Sikkim (Fig 1 and S2 Table). The DR increased in the successive rounds of AHS in all the nine states with no specific pattern emerging by each state (Table 4). The DR increase in the states of Chhattisgarh and Rajasthan was nearly 4 times high between AHS baseline and round 1 than between AHS rounds 1 and 2, while an opposite pattern was seen in Odisha.

**Table 3:** Comparison of disability rates for India between the data sources.

Type of disability	Disability rate per 100,000 persons (95% confidence interval)*		
	Census 2011 (% of total)	Household survey 2012–13^†^ (% of total)	Percent difference between the Census and household survey
**Overall**	**2,241.9 (2,241.1–2,242.8)**	**1,791.4 (1,785.9–1,796.9)**	**25.1**
Movement	451.9 (451.5–452.3) (20.2%)	562.5 (559.4–565.6) (31.4%)	-19.7
Visual	421.4 (421.0–421.8) (18.8%)	345.8 (342.4–348.2) (19.3%)	21.9
Hearing	425.9 (425.5–426.3) (19.0%)	188.9 (187.1–190.7) (10.5%)	125.5
Mental	183.9 (183.6–184.2) (8.2%)	247.4 (245.3–249.4) (13.8%)	-25.7
Speech^‡^	169.9 (169.7–170.1) (7.6%)	110.2 (108.8–111.5) (6.1%)	54.2
Multiple^§^	176.0 (175.7–176.2) (7.8%)	159.5 (157.9–161.2) (8.9%)	10.3
Other^ǁ^	412.8 (412.4–413.2) (18.4%)	177.2 (175.4–178.9) (9.9%)	133.0

*Data not available in the household survey for Delhi, Gujarat, Jammu and Kashmir, Dadra and Nagar Haveli, Daman and Dui, and Lakshadweep.

^†^Includes pooled data from the District Level Household Survey-4 (2012–13) and Annual Health Survey 2^nd^ updation round (2012–13).

^‡^Only for ages 3 years and above.

^§^Two or more disabilities.

^ǁ^A disability which is not covered under any of the above categories.

### Movement disability

Both the data sources considered movement disability irrespective of the use of a walking aid (S1 Table). The survey clearly states that any temporary limitation in the movement was not classified as movement disability, however this is not very clear in the Census. The Census definition included example of routine movement activities but the survey did not.

**Fig 2:**
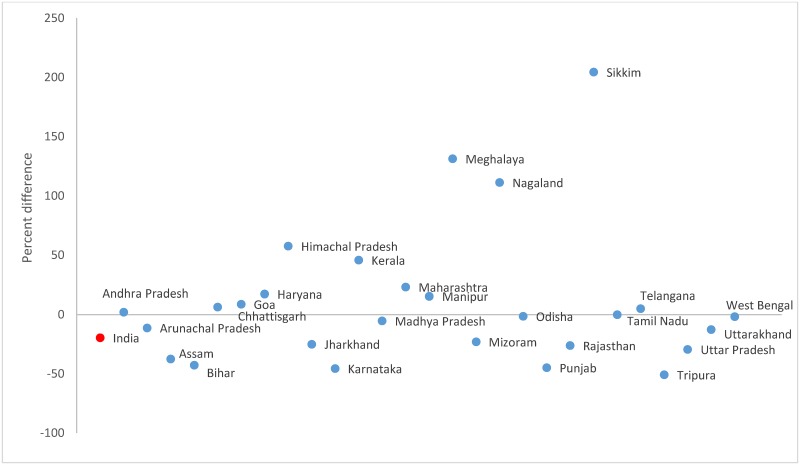
Percent difference in the movement disability rates between the Census 2011* and household survey 2012–2013^†‡^ for each Indian state. *Andhra Pradesh and Telangana were separated by the districts in the Census 2011. ^†^Includes pooled data from the District Level Household Survey-4 (2012–13) and Annual Health Survey 2^nd^ updation round (2012–13). ^‡^Data not available in the household survey for Delhi, Gujarat, Jammu and Kashmir, Dadra and Nagar Haveli, Daman and Dui, and Lakshadweep.

The movement DR was 19.7% lower in the Census 2011 (451.9; 95% CI 451.5–452.3) than the household survey (562.5; 95% CI 559.4–565.6, Table 3). Movement disability accounted for 20.2% and 31.4% of all disability in the Census and surveys, respectively. The difference in movement DR between the sources ranged from -51% in Tripura to 204% in Sikkim (Fig 2 and S2 Table). The movement DR increased across the successive rounds of AHS in all states except Uttar Pradesh and Uttarakhand (Table 4). The movement DR increased more between the AHS baseline and round 1 than between the AHS rounds 1 and 2 in the states of Assam (17 times) and Chhattisgarh (4 times) while an opposite pattern was seen in Odisha (6 times).

**Table 4:** Comparison of disability rates by state within the subsequent rounds of the Annual Health Survey.

Disability type	State	Disability rate per 100,000 person (95% confidence interval)			Percent change from Baseline to Round 1	Percent change from Round 1 to Round 2
		AHS 2010–11 (Baseline)	AHS 2011–12 (Round 1)	AHS 2012–13 (Round 2)		
**Overall**	Assam	1429.9 (1412.3–1447.4)	1506.6 (1488.6–1524.6)	1580.4 (1562.2–1598.6)	5.4	4.9
	Bihar	1308.4 (1295.8–1320.9)	1578.1 (1564.4–1591.9)	1871.3 (1855.6–1886.9)	20.6	18.6
	Chhattisgarh	1172.1 (1153.0–1191.2)	1674.6 (1652.0–1697.2)	1837.8 (1814.4–1861.2)	42.9	9.7
	Jharkhand	1350.2 (1334.2–1366.3)	1623.6 (1606.0–1641.2)	1884.3 (1864.0–1904.7)	20.2	16.1
	Madhya Pradesh	1395.0 (1379.9–1410.2)	1577.1 (1561.1–1593.0)	1723.7 (1707.2–1740.2)	13.1	9.3
	Odisha	1779.5 (1760.9–1798.1)	1843.5 (1824.6–1862.4)	2104.6 (2084.7–2124.5)	3.6	14.2
	Rajasthan	1393.9 (1376.9–1410.9)	1977.5 (1957.5–1997.6)	2159.9 (2138.8–2180.9)	41.9	9.2
	Uttar Pradesh	1213.1 (1203.1–1223.1)	1277.0 (1267.1–1286.9)	1322.9 (1312.9–1333.0)	5.3	3.6
	Uttarakhand	1227.7 (1210.9–1244.4)	1293.2 (1276.7–1309.7)	1341.6 (1324.7–1358.4)	5.3	3.7
**Movement**	Assam	325.5 (317.1–334.0)	385.7 (376.6–394.9)	390.0 (380.9–399.1)	18.5	1.1
	Bihar	499.5 (491.7–507.3)	548.0 (539.9–556.2)	620.1 (611.1–629.2)	9.7	13.2
	Chhattisgarh	484.0 (471.7–496.3)	650.4 (636.2–664.5)	701.3 (686.8–715.9)	34.4	7.8
	Jharkhand	412.8 (403.9–421.7)	522.6 (512.6–532.7)	598.3 (586.8–609.8)	26.6	14.5
	Madhya Pradesh	552.5 (543.0–562.0)	567.6 (557.9–577.2)	589.4 (579.7–599.1)	2.7	3.8
	Odisha	383.3 (374.6–392.0)	415.3 (406.2–424.3)	628.6 (617.6–639.5)	8.3	51.4
	Rajasthan	613.8 (602.5–625.2)	900.4 (886.8–914.0)	844 (831.0–857.6)	46.7	-6.3
	Uttar Pradesh	475.7 (469.4–481.9)	479.4 (473.3–485.5)	481.0 (474.9–487.0)	0.8	0.3
	Uttarakhand	536.6 (525.5–547.7)	503.1 (492.8–513.4)	420.1 (410.6–429.6)	-6.2	-16.5
**Visual**	Assam	283.2 (275.3–291.0)	292.0 (284.1–300.0)	295.1 (287.2–303.1)	3.1	1.1
	Bihar	230.8 (225.5–236.1)	298.9 (292.9–304.9)	357.7 (350.8–364.6)	29.5	19.7
	Chhattisgarh	180.9 (173.4–188.4)	264.1 (255.1–273.2)	281.7 (272.4–290.9)	46.0	6.7
	Jharkhand	260.2 (253.1–267.2)	291.0 (283.5–298.5)	321.3 (312.8–329.8)	11.8	10.4
	Madhya Pradesh	291.4 (284.4–298.3)	336.1 (328.7–343.5)	420.7 (412.5–428.9)	15.3	25.2
	Odisha	480.6 (470.9–490.4)	467.4 (457.8–477.0)	413.1 (404.2–422.0)	-2.7	-11.6
	Rajasthan	292.0 (284.1–299.8)	330.1 (321.9–338.4)	499 (489.3–509.7)	13.0	51.2
	Uttar Pradesh	233.0 (228.7–237.4)	213.5 (209.4–217.6)	188.6 (184.8–192.4)	-8.4	-11.7
	Uttarakhand	164.7 (158.6–170.9)	163.0 (157.1–168.9)	146.0 (140.4–151.6)	-1.0	-10.4
**Hearing**	Assam	220.9 (214.0–227.8)	213.8 (206.9–220.6)	220.5 (213.7–227.4)	-3.2	3.1
	Bihar	104.4 (100.8–108.0)	138.1 (134.0–142.2)	184.3 (179.3–189.2)	32.3	33.5
	Chhattisgarh	108.8 (103.0–114.7)	161.3 (154.2–168.3)	204.9 (197.1–212.8)	48.3	27.0
	Jharkhand	136.1 (131.0–141.3)	158.1 (152.6–163.7)	180.7 (174.4–187.1)	16.2	14.3
	Madhya Pradesh	155.1 (150.1–160.2)	180.6 (175.2–186.0)	186.9 (181.4–192.4)	16.4	3.5
	Odisha	357.2 (348.8–365.6)	383.4 (374.7–392.0)	267.5 (260.3–274.6)	7.3	-30.2
	Rajasthan	83.5 (79.3–87.7)	190.9 (184.6–197.2)	213 (206.8–220.2)	128.6	11.6
	Uttar Pradesh	113.1 (110.0–116.2)	91.9 (89.2–94.6)	96.0 (93.3–98.7)	-18.7	4.5
	Uttarakhand	109.7 (104.7–114.8)	108.5 (103.7–113.3)	94.2 (89.7–98.7)	-1.1	-13.2
**Mental**	Assam	241.8 (234.5–249.0)	247.4 (240.1–254.8)	293.6 (285.7–301.5)	2.3	18.7
	Bihar	189.7 (184.9–194.5)	210.9 (205.8–215.9)	252.9 (247.1–258.7)	11.2	19.9
	Chhattisgarh	193.3 (185.6–201.1)	241.8 (233.2–250.4)	262.3 (253.4–271.2)	25.1	8.5
	Jharkhand	274.7 (267.4–282.0)	305.5 (297.8–313.2)	348.6 (339.7–357.4)	11.2	14.1
	Madhya Pradesh	203.7 (197.9–209.5)	220.9 (214.8–226.9)	226.2 (220.2–232.2)	8.4	2.4
	Odisha	233.1 (226.3–239.9)	212.9 (206.4–219.4)	355.4 (347.1–363.7)	-8.7	66.9
	Rajasthan	191.3 (184.9–197.6)	249.6 (242.4–256.8)	278.0 (270.4–285.7)	30.5	11.4
	Uttar Pradesh	189.6 (185.6–193.6)	188.1 (184.2–191.9)	155.6 (152.2–159.1)	-0.8	-17.3
	Uttarakhand	195.5 (188.7–202.2)	189.8 (183.5–196.2)	156.8 (151.0–162.6)	-2.9	-17.4
Speech*	Assam	151.6 (145.9–157.3)	149.0 (143.3–154.7)	172.4 (166.3–178.4)	-1.7	15.7
	Bihar	113.3 (109.6–117.0)	129.0 (125.0–132.9)	146.4 (142.0–150.9)	13.9	13.5
	Chhattisgarh	67.9 (63.3–72.6)	89.3 (84.1–94.6)	97.0 (91.5–102.4)	31.4	8.6
	Jharkhand	100.9 (96.5–105.4)	119.4 (114.6–124.2)	147.7 (141.9–153.4)	18.3	23.7
	Madhya Pradesh	59.2 (56.1–62.3)	70.8 (67.4–74.2)	69.4 (66.1–72.8)	19.6	-2.0
	Odisha	78.2 (74.2–82.1)	169.6 (163.9–175.4)	137.5 (132.3–142.6)	116.9	-18.9
	Rajasthan	62.6 (59.0–66.3)	101.3 (96.7–105.9)	86.9 (82.6–91.2)	61.8	-15.1
	Uttar Pradesh	86.3 (83.7–89.0)	81.9 (79.4–84.5)	78.7 (76.2–81.2)	-5.1	-3.9
	Uttarakhand	75.2 (71.0–79.4)	76.5 (72.5–80.5)	72.5 (68.6–76.5)	1.7	-5.2
**Multiple**	Assam	206.9 (200.2–213.6)	158.1 (152.2–164)	123.0 (117.9–128.1)	-23.6	-22.2
	Bihar	170.7 (166.1–175.2)	172.9 (168.4–177.5)	178.0 (173.2–182.9)	1.3	2.9
	Chhattisgarh	137.0 (130.5–143.6)	178.7 (171.3–186.1)	179.9 (172.6–187.3)	30.4	0.7
	Jharkhand	165.5 (159.8–171.2)	156.5 (151.0–162.0)	164.9 (158.8–170.9)	-5.4	5.4
	Madhya Pradesh	133.1 (128.4–137.8)	149.1 (144.1–154.0)	157.2 (152.2–162.2)	12.0	5.4
	Odisha	247.1 (240.1–254.1)	185.0 (178.9–191.0)	200.7 (194.5–206.9)	-25.1	8.5
	Rajasthan	150.7 (145.1–156.3)	158.0 (152.3–163.8)	161 (155.9–167.6)	4.8	1.9
	Uttar Pradesh	115.4 (112.3–118.5)	125.0 (121.9–128.1)	116.4 (113.5–119.4)	8.3	-6.9
	Uttarakhand	145.9 (140.1–151.7)	138.4 (133.0–143.8)	128.9 (123.7–134.2)	-5.1	-6.9
**Others**^†^	Assam		60.5 (56.9–64.2)	85.8 (81.5–90.0)		41.8
	Bihar		80.3 (77.2–83.4)	131.8 (127.6–136.0)		64.1
	Chhattisgarh		89.0 (83.7–94.2)	110.7 (104.9–116.4)		24.4
	Jharkhand		70.5 (66.8–74.2)	122.9 (117.7–128.2)		74.3
	Madhya Pradesh		52.0 (49.1–54.9)	73.9 (70.5–77.4)		42.1
	Odisha		9.9 (8.5–11.3)	101.9 (97.5–106.3)		929.3
	Rajasthan		47.1 (44.0–50.2)	75 (71.9–79.9)		59.2
	Uttar Pradesh		97.1 (94.4–99.9)	206.6 (202.6–210.6)		112.8
	Uttarakhand		113.9 (109.0–118.8)	323.0 (314.7–331.3)		183.6

*Only for ages 3 years and above.

^†^This category was not available in the Annual Health Survey Baseline (2010–11).

### Visual disability

In both the data sources (S1 Table), individuals were considered with visually disabled based on their presenting vision (with corrective measure if in use). Furthermore, in the Census it is mentioned that the respondents were asked to count fingers from a distance of 10 feet in good day light to ascertain blurred vision but it is not clear if this was performed on all household members or only those who complained of visual disability. The Census also states that persons with night blindness or colour blindness only were not considered as having visual disability while the surveys do not mention anything specifically. One-eyed people were not considered as having visual disability in either source.

**Fig 3:**
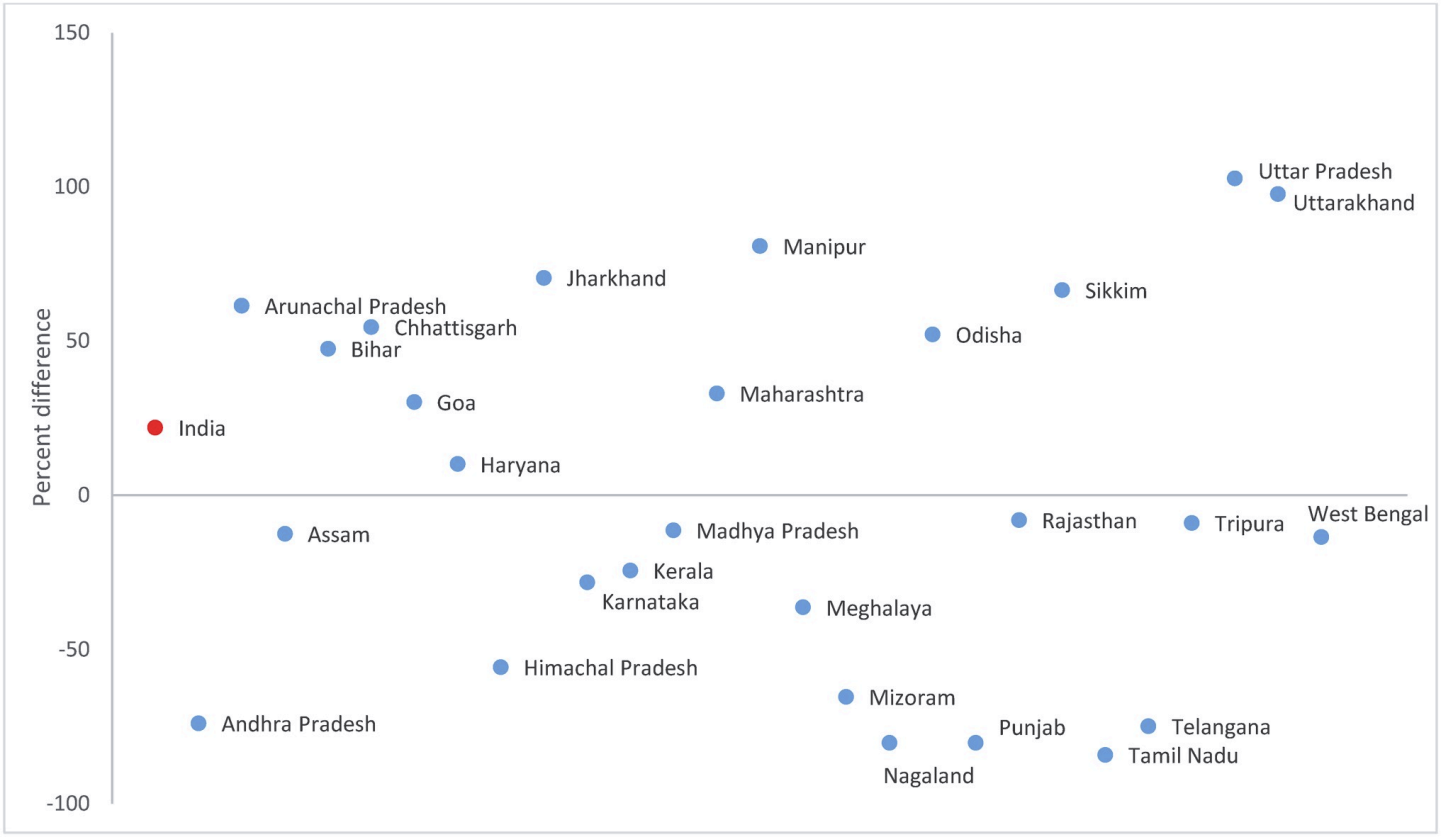
Percent difference in visual disability rate between the Census 2011* and household survey 2012–2013^†‡^ for each Indian state. *Andhra Pradesh and Telangana were separated by the districts in the Census 2011. ^†^Includes pooled data from the District Level Household Survey-4 (2012–13) and Annual Health Survey 2^nd^ updation round (2012–13). ^‡^Data not available in the household survey for Delhi, Gujarat, Jammu and Kashmir, Dadra and Nagar Haveli, Daman and Dui, and Lakshadweep.

The visual DR was estimated to be 21.9% higher in the Census (421.4; 95% CI 421.0–421.8 than the household survey (345.8; 95% CI 342.4–348.2, Table 3). Visual disability accounted for 18.8% and 19.3% of all disability in the Census and survey, respectively. The difference in visual DR between the sources ranged from -84% in Tamil Nadu to 103% in Uttar Pradesh followed by Uttarakhand (98%) (Fig 3 and S2 Table). Visual DR declined more between AHS rounds 1 and 2 than between AHS baseline and round 1 (Table 4) in Odisha (-11.6% vs -2.7%), Uttar Pradesh (-11.7% vs -8.4%), and Uttarakhand (-10.4% vs -1.0%). In the remaining AHS states, visual DR increased across successive rounds and the range of increase varied substantially from Chhattisgarh (6.9 times) to Bihar (1.5 times).

### Hearing disability

Both the data sources considered hearing disability irrespective of the use of hearing aid, and persons with hearing disability in one ear were not considered as having disability in either data source (S1 Table). A person born with hearing disability who was also unable to speak (deaf and mute) was considered as having multiple disabilities in the Census; the survey does not categorically mention anything about persons who were both deaf and mute.

**Fig 4:**
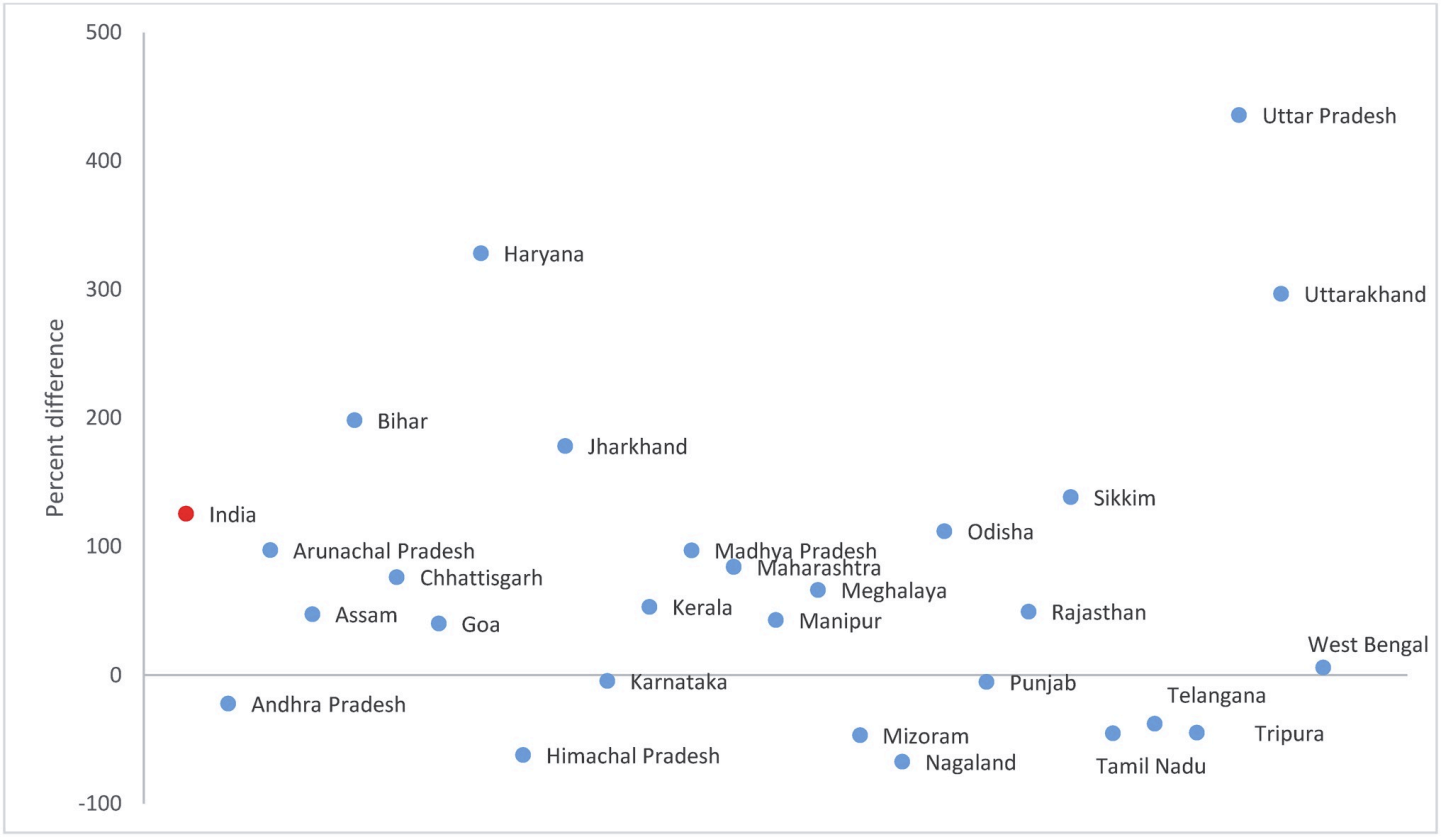
Percent difference in hearing disability rate between the Census 2011* and household survey 2012–2013^†‡^ for each Indian state. * Andhra Pradesh and Telangana were separated by the districts in the Census 2011. ^†^ Includes pooled data from the District Level Household Survey-4 (2012–13) and Annual Health Survey 2^nd^ updation round (2012–13). ^‡^ Data not available in the household survey for Delhi, Gujarat, Jammu and Kashmir, Dadra and Nagar Haveli, Daman and Dui, and Lakshadweep.

The hearing DR was 125.5% higher in the Census (425.9; 95% CI 425.5–426.3) than the survey (188.9; 95% CI 187.1–190.7, Table 3). Hearing disability accounted for 19.0% and 10.5% of the total disability in the Census and survey, respectively. The difference in hearing DR between the sources ranged from -67% in Nagaland to 436% in Uttar Pradesh (Fig 4 and S2 Table). The hearing DR declined between AHS baseline and round 1 in Uttar Pradesh (18.7%), while it declined between AHS rounds 1 and 2 in Odisha (-30.2%) and Uttarakhand (-13.2%) (Table 4). The increase in hearing DR was more between AHS baseline and round 1 than AHS rounds 1 and 2 in Rajasthan (128.6% vs 11.6%) Madhya Pradesh (16.4% vs 3.5%), and Chhattisgarh (48.3% vs 27.0%).

### Mental disability

The Census definition of mental disability was more comprehensive than the survey though both the data sources aimed to cover mental retardation and mental illness (S1 Table). The survey did not differentiate between mental retardation and mental illness for documentation whereas the Census did. The survey definition stated that persons who show sign of mental fatigue or dependency on others due to old age will not be considered as having mental disability, whereas it appears that the Census may have included them under this category of disability. It is interesting to note that the Census clearly states that people/students who are slow learners or with delayed development are not necessarily mentally retarded.

**Fig 5:**
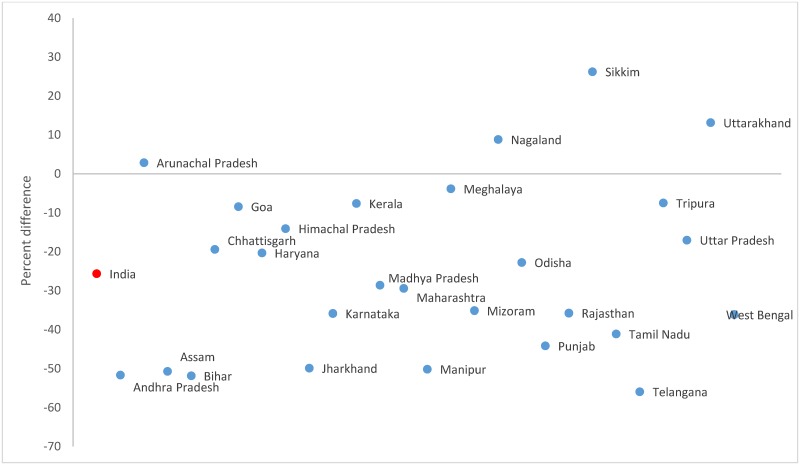
Percent difference in mental disability rate between the Census 2011* and household survey 2012–2013^†‡^ for each Indian state. * Andhra Pradesh and Telangana were separated by the districts in the Census 2011. ^†^ Includes pooled data from the District Level Household Survey-4 (2012–13) and Annual Health Survey 2^nd^ updation round (2012–13). ^‡^ Data not available in the household survey for Delhi, Gujarat, Jammu and Kashmir, Dadra and Nagar Haveli, Daman and Dui, and Lakshadweep.

The mental DR was 25.7% lower in the Census (183.9; 95% CI 183.6–184.2) than the survey (247.4; 95% CI 245.3–249.4; Table 3). Mental disability accounted for 8.2% and 13.8% of all disability in the Census and survey, respectively. Across the states, the percent difference in mental DR between data sources ranged from -56% in Telangana to 26% in Sikkim (Fig 5 and S2 Table). Within the AHS rounds, mental DR increased in all states except Uttarakhand and Uttar Pradesh (Table 4). The increase in mental DR was higher between AHS baseline and round 1 than between AHS rounds 1 and 2 in Chhattisgarh (3 times), Madhya Pradesh (3.5 times) and Rajasthan (2.7 times), while it was higher in rounds 1 and 2 in Odisha (8 times), Assam (8 times) and Bihar (1.8 times).

### Speech disability

Speech disability was assessed for persons aged 3 years and more in both the sources. The definition of speech disability was more or less similar between the two sources with the only difference being that the survey also included articulation defects along with stammering (S1 Table).

**Fig 6:**
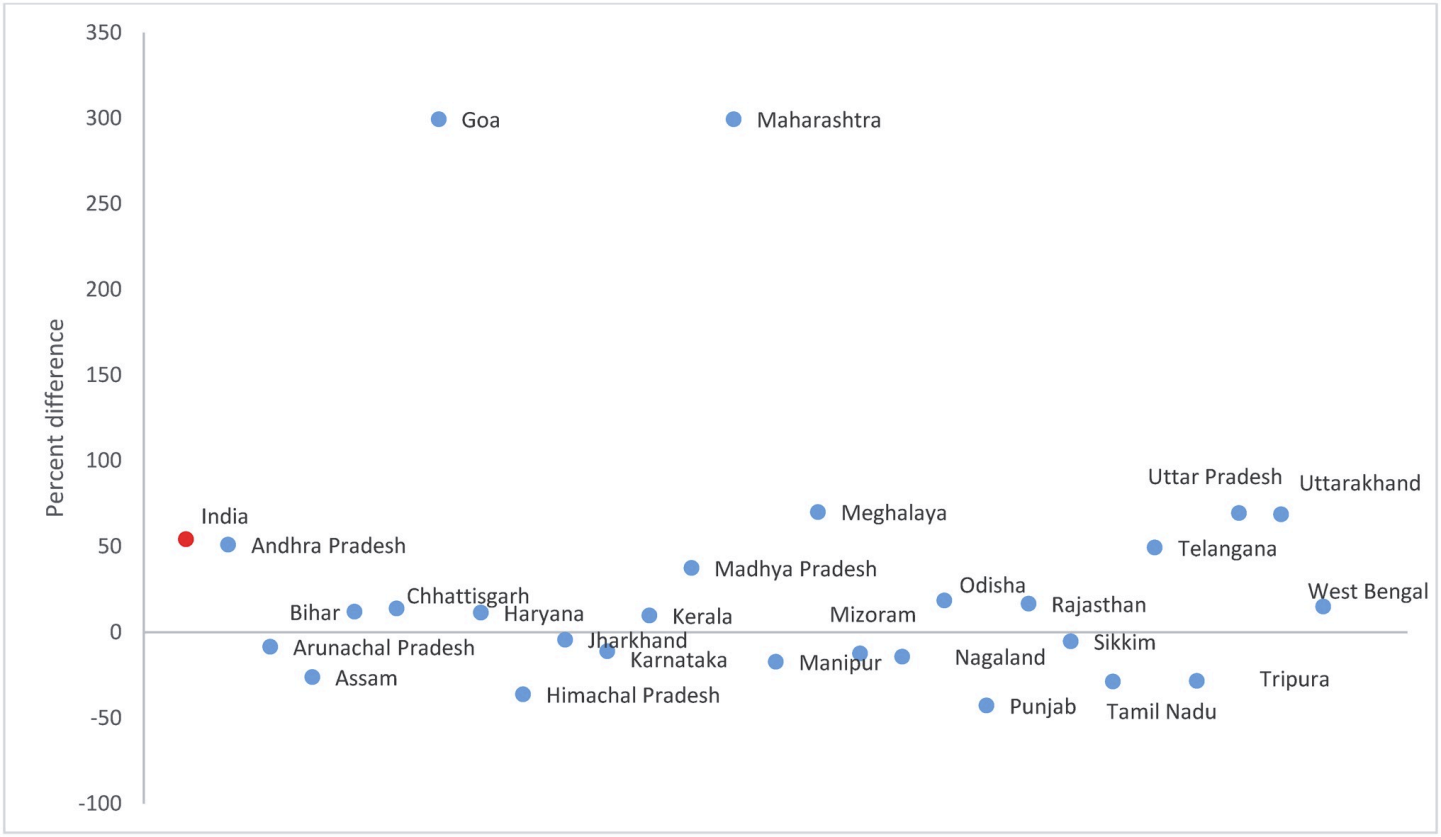
Percent difference in speech* disability rate between the Census 2011^†^ and household survey 2012–2013^‡ §^ for each Indian state. *Only for ages 3 years and above. ^†^Andhra Pradesh and Telangana were separated by the districts in the Census 2011. ^‡^ Includes pooled data from the District Level Household Survey-4 (2012–13) and Annual Health Survey 2^nd^ updation round (2012–13). ^§^ Data not available in the household survey for Delhi, Gujarat, Jammu and Kashmir, Dadra and Nagar Haveli, Daman and Dui, and Lakshadweep.

The speech DR was higher in the Census 2011 (169.9; 95% CI 169.7–170.1) by 54.2% than the surveys (110.2; 95% CI 108.8–115.5, Table 3). Speech disability accounted for 7.6% and 6.1% of the total disability in the Census and survey, respectively. The difference in speech disability between the sources ranged from -43% in Punjab to 300% in Maharashtra (Fig 6 and S2 Table). The speech DR increased between AHS baseline and round 1 (Table 4) and the range of increase varied substantially from Bihar (13.9%) to Odisha (116.9%). Between the AHS rounds 1 and 2, the speech DR declined in Odisha (-18.9%) and Rajasthan (-14.2%).

### Multiple disability

The definition of multiple disability was similar in the Census and survey (S1 Table). The multiple DR was 10.3% higher in the Census (176.0; 95% CI 175.7–176.2) than the survey (159.5; 95% CI 157.9–161.2; Table 3). The difference in multiple DR between the sources ranged from -76% in Tamil Nadu to 198% in Kerala (S1 Fig and S2 Table). Between AHS baseline and round 1 (Table 4), the multiple DR increased in Chhattisgarh (30.4%), Madhya Pradesh (12.0%) and Uttar Pradesh (8.3%) but declined in Odisha (-25.1%) and Assam (-23.6%). The multiple DR increased in Odisha (8.5%) but declined in Assam (-22.2%) and Uttar Pradesh (-6.9%) between AHS rounds 1 and 2.

### Other disability

A person with autism was considered as having other disability in Census while the survey does not mention this specifically (S1 Table). The other DR was 133% higher in the Census (412.8; 95% CI 412.4–413.2) than the surveys (177.2; 95% CI 175.4–178.9; Table 3). The difference in other DR between the sources ranged from -77% in Tamil Nadu to 450% in Madhya Pradesh (S2 Fig and S2 Table). Across all AHS states there was significant increase in other DR between AHS rounds 1 and 2 and ranged from 929% in Odisha to 24% in Chhattisgarh (Table 4).

## Discussion

We found the overall disability estimate for India to be 25.1% higher in the Census than in the household survey, and the rates for all types of disabilities except for the movement and mental disabilities were higher in the former than the latter. The proportion contribution of the different types of disabilities to the overall disability rate also varied between the data sources. The significant variations in the rates by state between the data sources and between the AHS rounds further highlighted the issues with the reliability of these estimates.

Though the difference in the overall DR was only 25% between the Census and household survey, this difference for hearing DR was 125% and that for mental and speech DR was -26% and 54%, respectively. At the state-level, the variations in disability-specific estimates ranged from -84% to 450%. As the time gap between data collection for the two sources was a maximum of 3 years, such large variations in the disability-specific rates are unexplainable only based on real change over time in the number of people experiencing disability. Similarly, we found wide variations in the yearly estimates of disability in the successive rounds of AHS which used the same definition of disability with neither migration nor actual change over time is likely to explain the extent of these variations seen ranging from -25% to 929% at the state-level across the AHS rounds. We discuss the potential reasons for these variations based on the review undertaken for this assessment.

With nearly similar definitions and operationalisation of the disability questions in the field in both the data sources, one would have expected similar rates across the sources. However, the Census recorded a higher rate than the survey for visual (22%), speech (54%), and hearing (125%) DR, whereas the survey recorded higher rates for movement (20%) and mental (26%) DR. This range of the variations in DR suggests further issues with the documentation of disability despite nearly similar definitions. We took a deep dive into the visual and hearing disabilities and compared these with the GBD Study estimates for the year 2011 to assess the validity of these estimates [35]. The visual DR in the Census was 421 per 100,000 population and was 345.8 in the survey with nearly similar definition of visual disability, except that the Census mentions checking for blurred vision but the process for it is not entirely clear. The GBD Study reported blindness prevalence estimate for India at 803 people per 100,000 population in 2011, which is nearly double of that reported as visual disability in the Census 2011. Furthermore, the GBD Study estimated severe vision loss prevalence at 762 people per 100,000 population in addition to blindness. The hearing DR was reported at 183.9 and 247.4 per 100,000 population in the Census and survey, respectively; and the GBD study estimated the prevalence rate of hearing loss (including severe, profound and complete hearing loss) at 514 people per 100,000 population [35]. In addition to these, moderate hearing loss prevalence in the GBD study was estimated at 2,704 people per 100,000 population. The Census and surveys that are not disability-focused are known to underestimate the true prevalence of disability because of limited space and time for any specific topic, or insufficient scope to collect data on different dimensions of disability [36–38]. Furthermore, this comparison of estimates with the GBD study may also highlight underestimation due to the absence of disease/condition-specific methodology in these sources. For example, the visual DR estimates for GBD study are derived from population-based blindness and visual impairment surveys and health records in India with standard clinical definitions of blindness and visual impairment based on eye examination [39], whereas the Census and DLHS/AHS surveys are based mainly on the proxy reporting of disability. Despite the evidence of varied reporting by proxy method in the health domain [40–45], most large-scale surveys and the Censuses use the proxy method to document disability based on the assumption that one household adult member will provide the same information as his/her household member, which may result in underestimation of disability [40, 46]. Recent evidence from India and Cameroon also suggests that there is less overlap between people who self-report a disability and those who are identified by clinical examination for visual, hearing and musculoskeletal functioning, and recommended measurement of disability at population level using both self-report and clinical examination [47]. Disability prevalence for adults 18 years or more of age has been previously reported at 24.9% for India from the World Health Survey, which again highlights the underestimation of disability prevalence in the two data sources.[29] The extent of variations in DR between the sources clearly suggest an urgent need to review the questions and methodology of data collection in the upcoming Census.

In addition to the above, we also found enormous state-wide variations in the rates of various disabilities between the Census and survey which could possibly be attributed to two reasons. First, the quality of data collection in the Census and surveys across the states could have resulted in measurement error. Several quality issues around data collection methods, skills and capacity of interviewers who collect data, and wording of questions have been highlighted for large-scale data collection in India, including the Census and household surveys, as a major reason for poor comparability of data across the various data sources [22, 48, 49]. For the Census 2011, absent or proxy interviewers who collected data on 1.2 billion people in just 20 days has been previously highlighted [22]. Second, these wide variations at the state-level may also reflect the way respondents interpret and respond to questions which may result in a systematic measurement error [14]. The social context and cultural circumstances around disability are known to influence it’s reporting [8]. For the Census 2011, underestimation of disability due to the social stigma despite the Census having run a campaign around accurately reporting disabilities has been previously highlighted [22]. Furthermore, the large variations seen in the yearly estimates of disability in the successive rounds of AHS raise further concerns about the validity and reliability of these estimates highlighting issues around how the questions were operationalized, how the respondents interpreted questions, how able and willing the respondents were to provide correct answers, and how accurately the answers were coded and classified [50]. Given these issues, the comparison of disability rates across the Indian states using these data may not be appropriate [37]. These findings suggest that prioritization is needed to better understand the validity and reliability of the questions used in the upcoming Census to document disability in India at the state-level to revise the questionnaire as necessary to obtain more robust disability estimates [51, 52].

Furthermore, in the Indian Rights of Persons with Disabilities Act (RPDA) 2016, persons with disabilities are categorised into three groups—with benchmark disability, with disability, and with disability having high support needs [18]. It is not clear how these categories are arrived at as neither of the two data sources collected data that allows for such details, thereby, further undermining the framing of policy and programs for people with disability in India [53]. The conceptual and definitional variability not only has an immediate impact on disability estimates, but may contribute in the long run to inconsistent or insufficient policy solutions and, may negatively impact the lives of those experiencing disability [54]. The WGSS approach has been incorporated into the United Nations principles and recommendations for the Censuses [55], and has been intensively tested in many countries [14, 56]. The WGSS relies on self-reporting wherein the respondent is not required to term him/herself as having disability, and participation restriction is documented related to seeing, hearing, locomotion, mental function, self-care and communication [16]. This approach does not use the term ‘disability’ in attempting to estimate the disability prevalence, given how stigma filled it may be and given the multitude of meanings people may attribute to this word [7]. In 2016, the WGSS was designated as the preferred method to use with the SDGs to number the world’s population of people with a disability by a group of leading UN agencies, civil society actors, and independent experts [57]. It is important to note that despite the several limitations of the WGSS including difficulty in developing questions that can elicit internationally comparable data [7, 57], it is a validated tool that can provide a quick, effective, and inexpensive way to generate disability data for governments, civil society, and research [57]. The WGSS has not been widely used in India. Two studies with relatively small sample sizes have highlighted issues related to documenting accurate disability data with the WGSS, and have called for the need for testing of the WGSS in India at a larger scale [47, 58]. With the extent of underestimation, validity and reliability issues highlighted in the disability estimates of Census 2011, the Indian government should initiate a dialogue with the relevant stakeholders within and external to the government to deliver estimates that are globally comparable and locally relevant to inform program planning for people with disabilities [38, 59]. Importantly, the context provided to the respondent for operationalisation of the questions for data collection also needs to be considered carefully to ensure that the prevalence is neither under or over-estimated [7].

In conclusion, with India having ratified the CRPD and being committed to SDGs, there is an urgent need to improve the disability estimates as well as an opportunity in the upcoming decennial Census of 2021, which India cannot afford to miss.

## Supporting information

S1 Table Definition of disability type in the Census 2011 and household surveys 2012–13. (DOCX)

S2 Table Comparison of disability rates for the Indian states between the Census 2011 and household surveys (HH survey) 2012–13. (DOCX)

S1 Fig Percent difference in multiple disability rate* between the Census 2011^†^ and household survey 2012–2013^‡ §^ for each Indian state. (DOCX)

S2 Fig Percent difference in other disability rate* between the Census 2011^†^ and household survey 2012–2013^‡ §^ for each Indian state. (DOCX)
