# Supplementary material for: India’s disability estimates: Limitations and way forward
Source: PLoS One. 2019 Sep 6;14(9):e0222159. doi: 10.1371/journal.pone.0222159 (PMC6730860; doi:10.1371/journal.pone.0222159)
Supplement: S1 Table — (DOCX) [file pone.0222159.s001.docx]

**S1 Table. Definition of disability type in the Census 2011 and household surveys 2012-13.**

| **Disability type** | **Census 2011** | **Household survey, 2012-13*** |
| --- | --- | --- |
| **Movement** | A person will be considered as having disability 'in movement' if she/he has a disability of bones, joints or muscles of the limbs leading to substantial restriction of movement. This would cover persons who:   - Do not have both arms or both legs; or - Are paralysed and are unable to move; or - Are unable to walk but crawl to move from one place to the other; or - Are able to move only with the help of caliper/s, wheelchair, tricycle, walking frame, crutches, etc; or - Have acute and permanent problems of joints/muscles that have resulted in limited movement; or - Have lost all the fingers or toes or a thumb; or - Are not able to move or pick up any small thing placed nearby; or - Have stiffness or tightness in movement; or - Have difficulty in balancing and coordinating body movements; or - Have loss of sensation in the body due to paralysis or leprosy or any other reason; or - Have any deformity of the body part/s like having a hunch back; or - Is very short statured (dwarf) - Manifestation of disability arising out of 'Cerebral Palsy' will be recorded as movement disability. | - Persons who are totally crippled (those who do not have both legs and hands or those without two legs or two hands) or persons who are paralyzed and are unable to move at all will be treated as having movement disability. - Persons with loss or absence of whole or part of hand or leg and persons suffering from inactivity of whole or part of body due to amputation, paralysis, deformity (including hunch back, deformed spine, etc) or dysfunction of limbs or joints which affected her/his `normal' ability to move (with or without aid) self or objects will also be considered as disabled. - Persons using artificial limbs will be treated as having movement disability. - Persons with temporary disability on the date of survey like stiff neck; back injury; fracture of hand(s), leg(s), etc. will not be treated as disabled. |
| **Visual** | A person will be considered as having disability 'in seeing' if she/he:   - Cannot see at all; or - Has no perception of light even with the help of spectacles; or - Has perception of light but has blurred vision even after using spectacles, contact lenses etc. A simple test is whether the person can count the fingers of hand from a distance of 10 feet in good daylight. Such persons can however, move independently with the help of remaining sight; or - Can see light but cannot see properly to move about independently; or - Has blurred vision but had no occasion to test if her/his eyesight would improve after taking corrective measures. - Persons with no vision in one eye but full vision in the other eye (one eyed persons) will not be considered as disabled in seeing. - Persons having night blindness alone will not be considered as disabled in seeing. - Persons having colour blindness alone will not be considered as disabled in seeing. | - Persons with no perception of light and those who perceive light but cannot see properly on account of low and/or blurred vision will be treated as suffering from visual disability. - Persons who suffer from low vision even after taking corrective measures (using spectacles, contact lenses etc), will also be considered as having visually disabled. - Persons who do not have difficulty in seeing after taking corrective measures, will not be treated as disabled. - Persons with proper vision in one eye (one eyed persons) will not be treated as disabled in seeing. |
| **Hearing** | A person will be considered as having disability 'in hearing' if she/he:   - Cannot hear at all; or - Has difficulty in hearing day-to-day conversational speech (hard of hearing); or - If she/he is using a hearing aid.   Persons having problem in only one ear will not be considered as having hearing disability. | - Persons who cannot hear at all and those having difficulty in hearing day to day conversational speech with or without the hearing aid will be classified as having hearing disability. - Persons having problem in only one ear will not be considered as having hearing disability. |
| **Mental** | Mental retardation: A condition of arrested or incomplete development of mind of a person which is specially characterized by sub-normality of intelligence. The onset of mental retardation is usually from birth or in some cases before the age of 18 years**.** A person will be considered as having the disability of 'mental retardation' if she/he:-   - Lacks understanding/comprehension as compared to her/his own age group; or - Was unable to communicate her/his needs when compared to other persons of her/his age group; or - Has difficulty in doing daily activities like looking after toilet needs, cleaning teeth, bathing, wearing clothes, taking care of personal hygiene and nutrition and general household tasks; or - Has difficulty in understanding routine instructions; or - Has extreme difficulty in making decisions, remembering things or solving problems. - All slow learners and persons with delayed development are not necessarily mentally retarded. - Students who are slow learners in school should definitely not be considered as mentally retarded.   No test is required to assess mental retardation. It should be left to the respondent to report whether the member of the household has mental retardation.  Mental illness: A person may be considered as having mental illness if she/he has a psychological or behavioural pattern associated with distress or disability that is not a part of normal development. The affected person is generally not able to cope with the problem. The onset or manifestation or realization of mental illness is not seen from birth unlike mental retardation. In general, a person may be considered as having the disability of 'mental illness' if she/he:   - Was taking medicines or other treatment for mental illness; or - Exhibits unnecessary and excessive worry and anxiety, unexplained withdrawal or problems in sleep, loss of appetite and/or depression, thought of dying, unattended personal hygiene; or - Exhibits repetitive (obsessive-compulsive) behaviour/thoughts; or - Exhibits sustained changes of mood or mood swings (joy and sadness) leading to having many days or weeks of not being able to function and behave normally; or - Has unusual experiences - such as hearing voices, seeing visions, experience of strange smells or sensations or strange taste; or - Exhibits unusual behaviours like talking/laughing to self, staring in space, excessive fear and suspicion without reason; or - Has difficulty in social interactions and adapting at home, at school, at workplace or generally in society. | - Persons who lack understanding appropriate to their age and have difficulty in carrying out the activities of daily routine like others of similar age such as communication (speech), self-care (brushing of teeth, wearing clothes, taking bath, taking food, personal hygiene etc.) will be treated as mentally disabled. - Those having problem in communicating and understanding verbal and non-verbal messages will be treated as mentally disabled. - The category of mentally disabled would include both mentally retarded as well as mentally ill and one need not try to distinguish between them. - Persons who show signs of mental fatigue, lack of understanding and are dependent on others for daily routine on account of being old, will not be considered as mentally disabled. |
| **Speech** | A person will be considered as having disability 'in speech', if she/he is above the age of 3 years and:   - Cannot speak at all or she/he is unable to speak normally on account of certain difficulties linked to speech disorder; or - Able to speak in single words only and is not able to speak in sentences; or - Stammers to such an extent that the speech is not comprehensible. - A person who is born with a hearing disability is also unable to speak (deaf mute). For the Census purpose this will be treated as a multiple disability. | - Presence of speech disability can be assessed only after the age of three years in children. - A person will be classified as having speech disability if she/he cannot speak at all or is unable to speak normally due to speech disorder. - Persons who speak in single words will be treated as having speech disability. - Persons having articulation defects and those who stammer will also be coded as having speech disability. - Persons who stammer but whose speech is comprehensible will not be classified as having speech disability. |
| **Multiple** | Persons suffering from any of the two or more disabilities listed in the question will be treated as having 'multiple disabilities'. The question has been designed to record a combination of maximum three types of disabilities as far as possible in the order of severity. | More than one disability described above. |
| **Other**[†](https://en.wikipedia.org/wiki/Dagger_(typography)) | - If the person has a disability that is not covered under any of the categories listed in the question. This category would include disabilities like Autism etc.; or - If the respondent fails to report the exact type of the disability. | Any other type of disability not covered above |

*Definition of disability in District Level Household Survey-4 (2012-13) was assumed to be similar to Annual Health Survey.

[†](https://en.wikipedia.org/wiki/Dagger_(typography))This category was not available in Annual Health Survey Baseline (2010-11).
