# Supplementary material for: India’s disability estimates: Limitations and way forward
Source: PLoS One. 2019 Sep 6;14(9):e0222159. doi: 10.1371/journal.pone.0222159 (PMC6730860; doi:10.1371/journal.pone.0222159)
Supplement: S2 Table — (DOCX) [file pone.0222159.s002.docx]

**S2 Table. Comparison of disability rates for the Indian states between the Census 2011 and household surveys (HH survey) 2012-13.**

| **State*/type of disability** | **Disability rate per 100,000 persons (95% confidence interval)** | | | | | | | | | | | | | | | |
| --- | --- | --- | --- | --- | --- | --- | --- | --- | --- | --- | --- | --- | --- | --- | --- | --- |
|  | **Overall** | | **Movement** | | **Visual** | | **Hearing** | | **Mental** | | **Speech**[†](https://en.wikipedia.org/wiki/Dagger_(typography)) | | **Multiple^‡^** | | **Other**^§^ | |
|  | **Census 2011^ǁ^** | **HH Survey^¶^** | **Census 2011^ǁ^** | **HH Survey^¶^** | **Census 2011^ǁ^** | **HH Survey^¶^** | **Census 2011^ǁ^** | **HH Survey^¶^** | **Census 2011^ǁ^** | **HH Survey^¶^** | **Census 2011^ǁ^** | **HH Survey^¶^** | **Census 2011^ǁ^** | **HH Survey^¶^** | **Census 2011^ǁ^** | **HH Survey** |
| **India** | 2241.9 (2241.1-2242.8) | 1791.4 (1785.9-1796.9) | 451.9 (451.6-452.3) | 562.5 (559.4-565.6) | 421.4 (421.0-421.8) | 345.8 (343.4-348.2) | 425.9 (425.6-426.3) | 188.9 (187.1-190.7) | 183.9 (183.7-184.2) | 247.4 (245.3-249.4) | 169.9 (169.7-170.2) | 110.2 (108.8-111.5) | 176.0 (175.7-176.2) | 159.5 (157.9-161.2) | 412.8 (412.4-413.2) | 177.2 (175.4-178.9) |
| Andhra Pradesh | 2974.4 (2968.8-2980.0) | 5210.1 (5059.1-5361.1) | 642.6 (639.9-645.2) | 630.7 (576.9-684.5) | 554.0 (551.6-556.5) | 2127.5 (2029.5-2225.6) | 477.0 (474.7-479.3) | 612.7 (559.7-665.7) | 208.5 (207.0-210.0) | 431.3 (386.8-475.8) | 272.2 (270.5-274.0) | 180.2 (151.4-209.0) | 211.3 (209.8-212.8) | 356.8 (316.3-397.3) | 608.8 (606.2-611.3) | 871.0 (807.8-934.1) |
| Arunachal Pradesh | 1932.0 (1909.1-1955.0) | 1316.9 (1235.8-1398.0) | 233.8 (225.7-241.8) | 263.6 (227.1-300.1) | 408.5 (397.8-419.1) | 253.1 (217.3-288.8) | 587.3 (574.6-600.1) | 297.9 (259.1-336.7) | 136.9 (130.8-143.1) | 133.1 (107.2-159.1) | 111.1 (105.6-116.7) | 121.3 (96.5-146.0) | 174.1 (167.1-181.0) | 93.6 (71.8-115.4) | 280.3 (271.4-289.1) | 154.2 (126.3-182.2) |
| Assam | 1538.4 (1534.1-1542.7) | 1580.4 (1562.2-1598.6) | 243.6 (241.8-245.3) | 390.0 (380.9-399.1) | 258.1 (256.4-259.9) | 295.1 (287.2-303.1) | 325.5 (323.5-327.5) | 220.5 (213.7-227.4) | 144.8 (143.5-146.2) | 293.6 (285.7-301.5) | 127.4 (126.1-128.6) | 172.4 (166.3-178.4) | 158.7 (157.3-160.1) | 123.0 (117.9-128.1) | 280.3 (278.4-282.1) | 85.8 (81.5-90.0) |
| Bihar | 2239.2 (2236.4-2242.1) | 1871.3 (1855.6-1886.9) | 355.0 (353.9-356.2) | 620.1 (611.1-629.2) | 527.5 (526.1-528.8) | 357.7 (350.8-364.6) | 549.6 (548.2-551.1) | 184.3 (179.3-189.2) | 121.8 (121.1-122.4) | 252.9 (247.1-258.7) | 164.1 (163.3-164.9) | 146.4 (142.0-150.9) | 106.5 (105.9-107.1) | 178.0 (173.2-182.9) | 414.7 (413.5-416.0) | 131.8 (127.6-136.0) |
| Chhattisgarh | 2446.4 (2440.4-2452.4) | 1837.8 (1814.4-1861.2) | 745.1 (741.7-748.4) | 701.3 (686.8-715.9) | 435.2 (432.6-437.7) | 281.7 (272.4-290.9) | 361.4 (359.1-363.7) | 204.9 (197.1-212.8) | 211.4 (209.6-213.2) | 262.3 (253.4-271.2) | 110.6 (109.3-111.9) | 97.0 (91.5-102.4) | 281.7 (279.6-283.7) | 179.9 (172.6-187.3) | 301.0 (298.9-303.2) | 110.7 (104.9-116.4) |
| Goa | 2263.4 (2239.2-2287.5) | 2181.6 (1894.5-2468.6) | 382.4 (372.4-392.5) | 351.9 (235.5-468.2) | 340.3 (330.9-349.8) | 261.4 (161.0-361.7) | 366.6 (356.8-376.4) | 261.4 (161.0-361.7) | 239.4 (231.5-247.3) | 261.4 (161.0-361.7) | 361.5 (351.7-371.2) | 90.5 (31.4-149.6) | 176.5 (169.7-183.4) | 221.2 (128.9-313.5) | 396.6 (386.4-406.8) | 733.9 (566.2-901.6) |
| Haryana | 2155.2 (2149.5-2160.9) | 1535.5 (1476.4-1594.6) | 457.7 (455.0-460.3) | 390.8 (360.8-420.8) | 326.2 (324.0-328.4) | 296.4 (270.3-322.5) | 455.7 (453.1-458.3) | 106.4 (90.7-122.1) | 182.5 (180.8-184.1) | 229.1 (206.1-252.0) | 85.9 (84.8-87.1) | 77.0 (63.6-90.3) | 186.4 (184.7-188.1) | 128.7 (111.4-145.9) | 460.8 (458.2-463.4) | 307.2 (280.6-333.8) |
| Himachal Pradesh | 2262.6 (2251.4-2273.7) | 4128.7 (3952.8-4304.6) | 474.2 (469.0-479.3) | 301.0 (252.6-349.4) | 379.9 (375.3-384.5) | 858.3 (776.7-939.8) | 389.0 (384.3-393.6) | 1027.1 (938.0-1116.2) | 206.2 (202.8-209.6) | 240.0 (196.7-283.2) | 120.6 (118.0-123.2) | 189.1 (150.7-227.6) | 270.0 (266.1-273.9) | 728.1 (653.0-803.3) | 422.8 (418.0-427.7) | 785.1 (707.1-863.1) |
| Jharkhand | 2334.1 (2329.0-2339.3) | 1884.3 (1864.0-1904.7) | 448.3 (446.0-450.6) | 598.3 (586.8-609.8) | 547.8 (545.3-550.4) | 321.3 (312.8-329.8) | 502.8 (500.4-505.2) | 180.7 (174.4-187.1) | 174.7 (173.2-176.1) | 348.6 (339.7-357.4) | 141.5 (140.2-142.8) | 147.7 (141.9-153.4) | 178.4 (176.9-179.8) | 164.9 (158.8-170.9) | 340.6 (338.7-342.6) | 122.9 (117.7-128.2) |
| Karnataka | 2167.4 (2163.8-2171.1) | 3078.7 (3008.2-3149.1) | 445.2 (443.5-446.8) | 819.9 (783.1-856.7) | 432.4 (430.7-434.0) | 602.6 (571.0-634.1) | 385.8 (384.2-387.3) | 403.9 (378.0-429.7) | 188.0 (187.0-189.1) | 293.1 (271.0-315.1) | 148.5 (147.6-149.5) | 166.7 (150.0-183.3) | 163.7 (162.7-164.7) | 401.3 (375.5-427.1) | 403.8 (402.2-405.4) | 391.3 (365.9-416.8) |
| Kerala | 2280.6 (2275.5-2285.6) | 2100.5 (1998.8-2202.3) | 513.8 (511.3-516.2) | 352.5 (310.4-394.5) | 345.8 (343.8-347.8) | 457.3 (409.5-505.2) | 315.4 (313.5-317.3) | 205.7 (173.6-237.9) | 397.0 (394.9-399.1) | 429.8 (383.4-476.2) | 123.8 (122.6-125.0) | 112.7 (88.9-136.5) | 297.1 (295.2-298.9) | 99.6 (77.2-122.0) | 287.8 (285.9-289.6) | 442.9 (395.8-490.0) |
| Madhya Pradesh | 2136.9 (2133.5-2140.2) | 1723.7 (1707.2-1740.2) | 557.3 (555.6-559.0) | 589.4 (579.7-599.1) | 372.8 (371.4-374.2) | 420.7 (412.5-428.9) | 368.1 (366.7-369.5) | 186.9 (181.4-192.4) | 161.5 (160.6-162.5) | 226.2 (220.2-232.2) | 95.5 (94.7-96.2) | 69.4 (66.1-72.8) | 175.4 (174.5-176.4) | 157.2 (152.2-162.2) | 406.2 (404.8-407.7) | 73.9 (70.5-77.4) |
| Maharashtra | 2637.1 (2634.1-2640.0) | 1858.8 (1806.2-1911.4) | 488.0 (486.7-489.3) | 396.6 (372.2-421.1) | 510.8 (509.5-512.2) | 384.0 (359.9-408.1) | 421.2 (420.0-422.4) | 228.7 (210.1-247.4) | 194.9 (194.0-195.7) | 276.2 (255.7-296.6) | 421.5 (420.3-422.7) | 105.5 (92.8-118.1) | 146.2 (145.5-147.0) | 156.1 (140.7-171.4) | 454.5 (453.3-455.7) | 311.7 (290.0-333.4) |
| Manipur | 2050.1 (2033.7-2066.5) | 1705.0 (1579.5-1830.5) | 186.1 (181.1-191.1) | 161.4 (122.5-200.4) | 672.1 (662.6-681.6) | 371.8 (312.8-430.8) | 451.4 (443.6-459.2) | 315.6 (261.2-369.9) | 220.8 (215.3-226.2) | 442.8 (378.4-507.1) | 93.2 (89.6-96.7) | 112.5 (80.0-145.0) | 124.4 (120.3-128.5) | 63.6 (39.2-88.0) | 302.1 (295.8-308.5) | 237.3 (190.1-284.4) |
| Meghalaya | 1493.7 (1479.9-1507.5) | 1930.2 (1783.1-2077.4) | 179.0 (174.2-183.9) | 77.4 (47.7-107.2) | 235.3 (229.8-240.8) | 369.4 (304.5-434.3) | 416.4 (409.0-423.7) | 250.2 (196.8-303.7) | 157.5 (153.0-162.0) | 163.8 (120.6-207.1) | 91.2 (87.8-94.7) | 53.6 (28.9-78.4) | 120.5 (116.6-124.5) | 59.6 (33.5-85.7) | 293.8 (287.7-300.0) | 956.2 (852.1-1060.3) |
| Mizoram | 1381.7 (1359.8-1403.5) | 2160.2 (2015.7-2304.7) | 180.1 (172.2-188.0) | 234.0 (186.0-282.0) | 185.5 (177.4-193.5) | 534.9 (462.4-607.4) | 305.7 (295.4-316.0) | 573.5 (498.4-648.5) | 240.2 (231.0-249.3) | 370.3 (309.9-430.7) | 106.0 (99.9-112.1) | 120.9 (86.3-155.4) | 189.8 (181.7-198.0) | 100.3 (68.8-131.8) | 174.4 (166.6-182.3) | 226.3 (179.1-273.5) |
| Nagaland | 1497.6 (1480.7-1514.6) | 3427.7 (3247.8-3607.5) | 193.5 (187.4-199.6) | 91.6 (61.7-121.5) | 209.8 (203.4-216.1) | 1066.2 (964.7-1167.8) | 451.9 (442.5-461.2) | 1384.3 (1268.8-1499.8) | 113.5 (108.8-118.2) | 104.3 (72.4-136.3) | 115.9 (111.2-120.7) | 134.9 (98.6-171.2) | 168.6 (162.9-174.3) | 371.5 (311.4-431.7) | 244.5 (237.6-251.4) | 274.8 (223.1-326.6) |
| Odisha | 2964.7 (2959.6-2969.8) | 2104.6 (2084.7-2124.5) | 619.2 (616.8-621.6) | 628.6 (617.6-639.5) | 628.5 (626.1-630.9) | 413.1 (404.2-422.0) | 566.7 (564.4-568.9) | 267.5 (260.3-274.6) | 274.5 (273.0-276.1) | 355.4 (347.1-363.7) | 163.2 (162.0-164.5) | 137.5 (132.3-142.6) | 300.7 (299.0-302.3) | 200.7 (194.5-206.9) | 411.9 (409.9-413.8) | 101.9 (97.5-106.3) |
| Punjab | 2357.5 (2351.9-2363.2) | 4604.0 (4501.9-4706.1) | 468.7 (466.2-471.3) | 850.0 (805.3-894.7) | 296.3 (294.3-298.3) | 1498.6 (1439.4-1557.8) | 528.8 (526.1-531.5) | 557.8 (521.5-594.1) | 241.5 (239.7-243.3) | 432.4 (400.5-464.4) | 88.5 (87.4-89.6) | 154.4 (135.3-173.6) | 136.9 (135.5-138.2) | 255.7 (231.1-280.3) | 596.9 (594.1-599.8) | 854.9 (810.1-899.8) |
| Rajasthan | 2281.2 (2277.6-2284.7) | 2159.9 (2138.8-2180.9) | 623.4 (621.6-625.3) | 844.3 (831.0-857.6) | 459.0 (457.4-460.6) | 499.5 (489.3-509.7) | 319.3 (318.0-320.6) | 213.5 (206.8-220.2) | 178.6 (177.6-179.6) | 278.0 (270.4-285.7) | 101.4 (100.6-102.1) | 86.9 (82.6-91.2) | 308.1 (306.8-309.4) | 161.8 (155.9-167.6) | 291.3 (290.0-292.6) | 75.9 (71.9-79.9) |
| Sikkim | 2978.7 (2936.0-3021.3) | 1440.6 (1266.5-1614.8) | 338.5 (324.0-353.1) | 111.2 (62.5-160.0) | 454.0 (437.1-470.9) | 272.6 (196.3-348.8) | 875.1 (851.7-898.4) | 367.1 (278.7-455.5) | 168.5 (158.2-178.8) | 133.5 (80.1-186.9) | 258.3 (245.5-271.0) | 272.6 (196.3-348.8) | 481.5 (464.1-498.9) | 194.7 (130.2-259.1) | 402.7 (386.8-418.6) | 89.0 (45.4-132.6) |
| Tamil Nadu | 1635.5 (1632.6-1638.4) | 4550.6 (4454.2-4647.1) | 398.1 (396.7-399.6) | 398.6 (369.4-427.8) | 176.6 (175.6-177.6) | 1117.7 (1069.1-1166.4) | 305.3 (304.0-306.5) | 556.9 (522.5-591.4) | 185.5 (184.5-186.5) | 315.0 (289.0-340.9) | 111.0 (110.2-111.8) | 155.5 (137.3-173.8) | 128.6 (127.8-129.4) | 543.0 (509.0-577.0) | 330.4 (329.1-331.7) | 1463.9 (1408.3-1519.5) |
| Telangana | 2469.9 (2465.5-2474.2) | 4223.3 (4061.8-4384.7) | 633.3 (631.1-635.5) | 603.6 (541.4-665.7) | 411.4 (409.6-413.2) | 1636.3 (1534.5-1738.1) | 337.0 (335.3-338.6) | 541.5 (482.6-600.4) | 206.9 (205.6-208.1) | 469.4 (414.6-524.3) | 250.5 (249.1-251.9) | 167.7 (134.8-200.5) | 234.9 (233.5-236.2) | 154.2 (122.7-185.7) | 395.9 (394.2-397.7) | 650.5 (586.0-715.0) |
| Tripura | 1751.4 (1738.0-1764.8) | 2469.3 (2241.9-2696.6) | 318.7 (312.9-324.4) | 648.0 (530.5-765.6) | 294.7 (289.2-300.3) | 324.0 (240.8-407.3) | 318.3 (312.6-324.1) | 575.4 (464.6-686.2) | 196.4 (191.9-200.9) | 212.3 (144.9-279.7) | 124.3 (120.7-127.9) | 173.2 (112.3-234.1) | 177.1 (172.8-181.4) | 162.0 (103.1-220.9) | 321.9 (316.1-327.7) | 374.3 (284.8-463.8) |
| Uttar Pradesh | 2080.7 (2078.7-2082.7) | 1322.9 (1312.9-1333.0) | 339.2 (338.4-340.0) | 481.0 (474.9-487.0) | 382.4 (381.5-383.2) | 188.6 (184.8-192.4) | 514.4 (513.4-515.4) | 96.0 (93.3-98.7) | 129.1 (128.6-129.6) | 155.6 (152.2-159.1) | 133.4 (132.9-133.9) | 78.7 (76.2-81.2) | 108.6 (108.2-109.1) | 116.4 (113.5-119.4) | 473.7 (472.7-474.6) | 206.6 (202.6-210.6) |
| Uttarakhand | 1836.9 (1828.6-1845.2) | 1341.6 (1324.7-1358.4) | 366.8 (363.1-370.5) | 420.1 (410.6-429.6) | 288.6 (285.3-291.9) | 146.0 (140.4-151.6) | 373.6 (369.8-377.4) | 94.2 (89.7-98.7) | 177.4 (174.8-180.0) | 156.8 (151.0-162.6) | 122.4 (120.3-124.6) | 72.5 (68.6-76.5) | 203.5 (200.7-206.3) | 128.9 (123.7-134.2) | 304.6 (301.2-308.0) | 323.0 (314.7-331.3) |
| West Bengal | 2210.2 (2207.2-2213.2) | 2033.0 (1951.9-2114.1) | 353.8 (352.6-355.0) | 360.3 (325.9-394.8) | 465.0 (463.6-466.4) | 538.4 (496.3-580.4) | 345.3 (344.1-346.5) | 325.9 (293.2-358.7) | 227.9 (226.9-228.9) | 356.9 (322.6-391.2) | 161.4 (160.6-162.2) | 140.2 (118.7-161.7) | 215.3 (214.3-216.2) | 128.1 (107.6-148.7) | 441.4 (440.1-442.8) | 183.2 (158.6-207.8) |

*Data not shown for Delhi, Gujarat, Jammu and Kashmir, Dadra and Nagar Haveli, Daman and Dui, and Lakshadweep as it was not available in the household survey.

[^†^](https://en.wikipedia.org/wiki/Dagger_(typography))Only for ages 3 years and above.

^‡^A combination of two or more disabilities listed in the question.

^§^ A disability that is not covered under any of the categories listed in the question.

^ǁ^ Andhra Pradesh and Telangana separated by the districts.

**^¶^** Includes pooled data from the District Level Household Survey-4 (2012-13) and Annual Health Survey 2^nd^ updation round (2012-13).

HH survey denotes household survey
