# Supplementary material for: India’s disability estimates: Limitations and way forward
Source: PLoS One. 2019 Sep 6;14(9):e0222159. doi: 10.1371/journal.pone.0222159 (PMC6730860; doi:10.1371/journal.pone.0222159)
Supplement: S2 Fig — (DOCX) [file pone.0222159.s004.docx]

**S2 Fig. Percent difference in other disability rate* between the Census 2011^†^ and household survey 2012-2013^‡ §^ for each Indian state.**

* A disability that is not covered under any of the categories listed in the question.

^†^Andhra Pradesh and Telangana were separated by the districts in the Census 2011.

^‡^ Includes pooled data from the District Level Household Survey-4 (2012-13) and Annual Health Survey 2^nd^ updation round (2012-13).

^§^ Data not shown for Delhi, Gujarat, Jammu and Kashmir, Dadra and Nagar Haveli, Daman and Dui, and Lakshadweep as it was not available in the household survey.
